# Inflammatory myofibroblastic tumor of the urinary bladder: a case report

**DOI:** 10.11604/pamj.2023.44.119.38156

**Published:** 2023-03-07

**Authors:** Hamza Dergamoun, Abdelaziz El Gdaouni, Adil Taoufik, Imad Ziouziou

**Affiliations:** 1 Department of Anatomy, Agadir University Hospital, Faculty of Medicine and Pharmacy, Ibn Zohr University, Agadir, Morocco,; 2 Department of Urology, Agadir University Hospital, Faculty of Medicine and Pharmacy, Ibn Zohr University, Agadir, Morocco

**Keywords:** Inflammatory myofibroblastic tumor, bladder, case report

## Abstract

The inflammatory myofibroblastic tumor (IMT) is a rare lesion, particularly in the urinary bladder. Inflammatory myofibroblastic tumor mainly affects children and young adults. It is unknown neoplastic potential, characterized by spindle cell proliferation with characteristic fibroinflammatory and pseudo-sarcomatous appearance. We describe a 36-year-old Moroccan man, who presented with hematuria the last week. The cystoscopy found a large bladder mass with necrotic-looking floating lesions, located in the trigonal area and left lateral wall on the dome of the urinary bladder. The patient underwent transurethral resection of the bladder tumor (TUR-BT). The histopathology and immunohistochemistry showed an IMT. No evidence of regrowth or residual tumor in 9 months of follow-up cystoscopy. In conclusion, even though, urinary bladder IMT is a rare occurrence, it is associated with a good prognosis. Histopathology investigation and immunohistochemistry analysis are essential to confirm the diagnosis. Complete TUR-BT is the treatment of choice.

## Introduction

Inflammatory myofibroblastic tumors (IMTs) are lesions classified by World Health Organization as intermediate-grade neoplasms [1]. They are a group of solid mesenchymal tumors that occur in young adults. They can affect a large number of organs, especially the lungs, but urinary bladder location is rare [2,3]. The pathogenesis remains poorly known, but recent studies of molecular genetics support neoplastic origin [3]. This report describes our experience with a case of IMT of the urinary bladder and reviews diagnostic and therapeutic features.

## Patient and observation

**Patient information:** a 36-year-old man, presented to the emergency department with a one-week history of total macroscopic hematuria associated with irritative voiding symptoms and pollakiuria. He mentioned a history of weight loss, decreased appetite, and generalized weakness. There was no significant medical or family history or any relevant past interventions.

**Clinical findings:** the physical examination was unremarkable. The temperature was 37.3°C, blood pressure 114/84 mmHg, and pulse rate regular at 76 beats/min. In palpation, we revealed mild tenderness in the hypogastric area but without any palpable mass.

**Figure 1:**
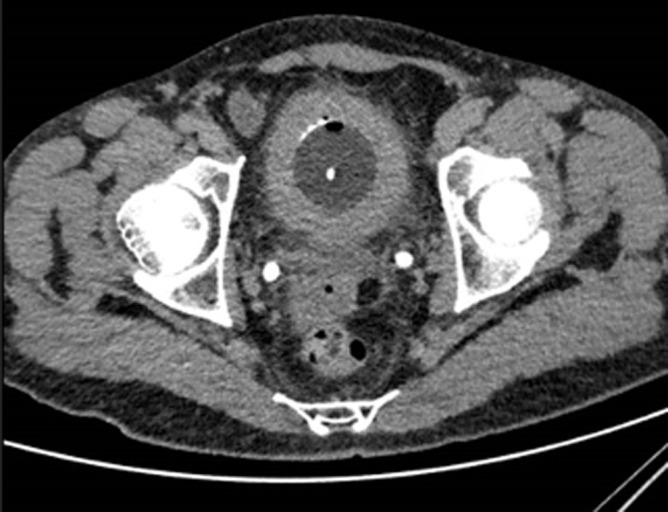
computed tomography scan showing a thick bladder wall

**Figure 2:**
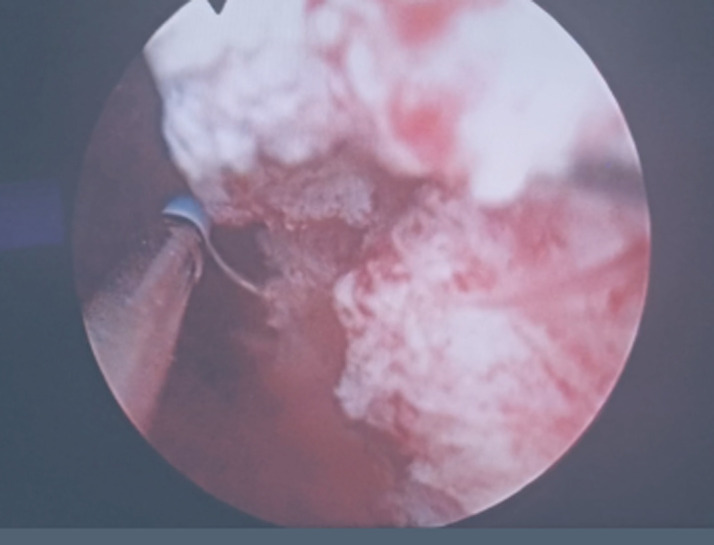
macroscopic aspect of the bladder mass located in the trigonal area and left lateral wall on the dome of the bladder

**Diagnostic assessment:** laboratory tests did not detect any alterations, and gave the following results: white blood cell count was 96.000/mm^3^, red blood cell count 4.3 x 106/mm^3^, hemoglobin 10.3 g/dL, hematocrit 33.1%, platelets 18.4 x 104/μL. Blood urea nitrogen 11.6 mg/dL, creatinine 8 mg/L, C-reactive protein 13 mg/L. A thoracoabdominal and pelvic CT scan revealed a thickening of the bladder wall around its entire circumference, heterogeneously enhancing after injection of the contrast medium without mass localization (Figure 1). The cystoscopy found a large bladder mass, located in the trigonal area and left lateral wall on the dome of the bladder, as well as necrotic-looking floating lesions (Figure 2).

**Therapeutic interventions:** the patient underwent a complete transurethral resection of the bladder tumor (TUR-BT) under general anesthesia. There were no post-operative complications.

**Figure 3:**
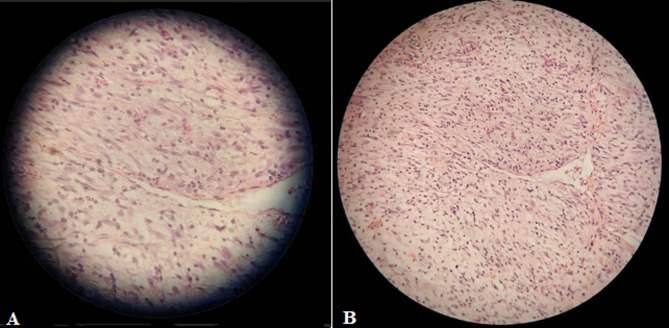
histological aspect of resected mass that widely infiltrated by spindle cells: A); in a myxoid stroma accompanied by an inflammatory cell infiltrate (H and E, x100); B); spindle myoepithelial cell proliferation (H and E, x400)

**Follow-up and outcome of interventions:** the postoperative course was without complications, and the patient was uneventfully discharged on the second postoperative day. Pathological assessment of the specimen showed a few spindle cells and inflammatory cells comprised of lymphocytes and plasma cells infiltrating the deep muscle, without necrotic tissue and with low mitotic activity (Figure 3). On immunohistochemistry, the tumor expressed Desmin, SMA, and ALK-1 and was immune-negative for cytokeratin and myogenin. The follow-up was carried out by a cystoscopy performed at 3 and 9 months after TUR-BT. The patient was asymptomatic and the cystoscopy demonstrated no evidence of regrowth or residual tumor.

**Patient perspective:** the patient was pleased with the care she received throughout therapy.

**Informed consent:** written informed consent was obtained from the patient for participation in our study.

## Discussion

Urinary bladder IMT is a rare tumor that has been identified in various organs, frequently on the mesentery, omentum, retroperitoneum, pelvis, and abdominal soft tissues in 73% of cases [1,3]. In the genitourinary tract, it can involve the kidneys, ureter, urethra, prostate, and testis, but most commonly seen in the urinary bladder [4,5]. The first case of urinary bladder IMT was reported by Roth in 1980 [6]. The pathogenesis remains little known. Several hypotheses have been raised, such as trauma, surgery, infection, and autoimmunity [2,7]. A chromosomal abnormality of chromosome 2p23 and cytogenetic clonality were found, consolidating the concept of a neoplastic origin [8]. Regarding molecular pathways, Lu *et al*. reported that Upstream frameshift 1 (UPF1) mutations downregulate nonsense-mediated RNA decay (NMD), leading to NF-κB (nuclear factor kappa light chain enhancer of activated B cells) overexpression, which contributes to the immune infiltration that is characteristic of IMT [9].

According to a systematic review of case series published up to 2013 involving 182 patients, IMT is slightly more common in women (51.7%). The average age of onset is 38± 16 years [10]. The clinical signs are not specific, painless gross hematuria is a common clinical manifestation and may associate with irritative symptoms or pelvic pain, as seen in our case [11]. Systemic manifestations are also possible but not usual [4]. In half of the cases, these tumors are not symptomatic [4]. There are no radiologic characteristics allowing to differentiate these tumors from malignant urothelial proliferation [12]. However, polypoid nodules on the bladder walls with ring enhancement on contrast-enhanced CT may be valuable in the diagnostic imaging of IMTs of the urinary system [13]. On cystoscopy, tumors are usually located in the upper or anterior wall of the bladder and usually appear as a budding, polypoid or nodular mass [2,14]. In our case, the computed tomography (CT) scan was inconclusive and the mass was detected in cystoscopy.

On histological examination, the presence of lymphocytic infiltrates and spindle myoepithelial cell proliferation remain the essential criteria for diagnosis [13]. The differential diagnosis is made with 3 types of malignancies: high-grade urothelial carcinoma with fusiform sarcomatoid variation, leiomyosarcoma, and embryonic rhabdomyosarcoma [5]. It is important to be able to demonstrate the myofibroblastic nature of these elements, which express muscle-specific actin and smooth muscle actin in immunohistochemistry and smooth muscle actin consistently and occasionally desmin, as seen in our case [13,15]. Treatment of IMT usually consists of TUR-BT or partial cystectomy because of its low risk of distant metastases [16]. Radical cystectomy has been reported but is not the treatment of choice. In our case, complete TUR-BT was successfully performed. The prognosis is difficult to establish. Nevertheless, the majority of cases reported have a favorable outcome, as mentioned in our case. Very rare cases of spontaneous regression have also been reported [6]. Cases of recurrence and one case of metastasis have been reported by Harik *et al*. [15].

## Conclusion

Urinary bladder inflammatory myofibroblastic tumors are rare, usually with a good prognosis. The pathogenesis remains poorly understood and controversial. Clinical signs are non-specific and the treatment is essentially complete surgical resection. The histological studies make it possible to diagnose and distinguish them from other malignant lesions, thus avoiding unnecessary radical surgery.
